# Intrapersonal Emotional Intelligence during Adolescence: Sex Differences, Connection with other Variables, and Predictors

**DOI:** 10.3390/ejihpe10030064

**Published:** 2020-09-18

**Authors:** Maite Garaigordobil

**Affiliations:** Faculty of Psychology, University of the Basque Country UPV/EHU, Avenida de Tolosa, 70, 20018 Donostia, Spain; maite.garaigordobil@ehu.eus

**Keywords:** emotional intelligence, personality, empathy, self-esteem, conflict resolution, parental style, bullying, cyberbullying, antisocial behavior

## Abstract

This study explores Intrapersonal Emotional Intelligence (IEI) with the objectives of: (1) analyzing possible differences due to sex and age, and the request for psychological assistance for behavioral and emotional problems; (2) finding evidence of personality traits, social behaviors, and parental socialization styles that are characteristic of adolescents with low IEI; and (3) identifying variables that predict high IEI. The sample comprised 2283 participants aged 12–17 years from the Basque Country (northern Spain). Results: (1) Females had greater emotional attention capacity but there were no sex differences in emotional comprehension and emotion regulation; (2) adolescents aged 12–14 showed higher scores in comprehension and emotion regulation than those aged 15–17; (3) adolescents who consulted a psychologist for problems (anxiety, depression, violence, etc.) had lower emotion regulation; (4) adolescents with low IEI had less empathy, self-esteem, extroversion, openness, agreeableness, and responsibility, and lower use of cooperative and passive conflict-resolution strategies, and their parents had a low level of acceptance-affection towards their children. They also engaged in more bullying/cyberbullying and antisocial behaviors. (5) High IEI predictor variables were: using cooperative conflict-resolution strategies; traits such as extroversion, responsibility, openness, and empathy; and a high level of maternal acceptance-affection. The work identifies relevant variables for designing intervention programs and shows the importance of promoting IEI and interpersonal emotional intelligence as a factor in the development and prevention of bullying/cyberbullying.

## 1. Introduction

### 1.1. Emotional Intelligence: Conceptualization

The idea of emotional intelligence (EI) has been present for many years but, in the last two decades, more research has been dedicated to this subject. Different theories have thus emerged and there is currently no single definition of the concept. In fact, research studying EI is divided into three groups according to their conception of this construct. EI can be understood as a skill, a trait, or something that unites skills and traits. According to this classification, the EI skill is a cognitive skill related to emotions. Mayer and Salovey [1] defined EI as the ability to perceive and express emotions, assimilate emotions into thought, understand and reason with emotions, and regulate emotions in oneself. In contrast, the EI trait has been defined as a set of emotional perceptions at the lowest levels of the personality hierarchy [2]. Petrides and Furnham [2] concluded that trait EI is a composite construct that belongs to the lower-order stratum of established personality taxonomies.

Finally, the mixed models argue that EI involves both cognitive-emotional skills and personality traits. As an example of this model, Goleman [3] defined EI as the capacity to recognize one’s own feelings, the feelings of others, and to motivate oneself to adequately deal with one’s relationships with others and with oneself. In addition, Bar-On [4] defined EI as a set of personal, emotional, and social factors influencing people’s ability to adapt to the demands and pressures of the environment. For further details on the models, a recent review of the models can be consulted [5]. 

The present work, which analyzes the variables of Intrapersonal Emotional Intelligence (IEI) evaluated with the TMMS-24, is underpinned by the model of Salovey and Mayer [6]. This model considers EI as an ability and defines it as a set of cognitive-emotional skills related to the management of emotions. EI consists of the ability to manage emotions, discriminate them, and use this knowledge to direct one’s thoughts and actions. It also refers to the ability to express and understand emotions and use them for personal growth. These skills are not always innate, so they can be learned and trained. The TMMS-24 measures attention, comprehension, and emotion regulation, and is one of the most extensively used instruments of this model [7].

Subsequently, Mayer and Salovey [1] broadened the definition of EI and considered that EI implies the ability to accurately perceive, value, and express emotions; the ability to access or generate feelings that facilitate thinking; the ability to understand emotions and emotional knowledge; and the ability to regulate emotions, promoting emotional and intellectual growth. Mayer and Salovey [8] structured EI as a model of four interrelated branches. (1) Emotional perception: The ability to pay attention to emotional signs of facial expression, body movements, and tone of voice. It refers to the accuracy with which people can identify their emotions and those of others, as well as the physiological and cognitive sensations they provoke. (2) Emotional facilitation of thought: the ability to consider feelings when reasoning or problem-solving. It refers to how emotions affect the cognitive system and how affective states influence decision-making. Emotions and moods modulate the person’s perspective, favoring the perception of various points of view. This ability implies that emotions act positively on reasoning and information processing. (3) Emotional comprehension: The ability to label emotions and recognize subgroups of feelings. Emotional comprehension involves understanding and analyzing emotions, using emotional knowledge. In addition, it implies the ability to know the causes and consequences of different emotions, and to interpret the meaning of complex emotions. (4) Emotion regulation: The ability to be open to and reflect on feelings (positive and negative), and to reject or take advantage of the information that accompanies feelings depending on their usefulness. This ability serves to regulate one’s own emotions and those of others, implementing various emotion-regulation strategies that modify feelings, neutralizing negative emotions and enhancing the positive ones. These four branches are hierarchically connected to each other, going from basic psychological processes to the most complex ones. Therefore, good emotion regulation requires good emotional perception, emotional facilitation, and emotional comprehension. For this reason, Pérez-González [8] referred to the model as the pyramidal EI model. 

### 1.2. Emotional Intelligence: Sex Differences and Age-Related Changes

The results on the existence and magnitude of sex differences in EI have been contradictory. A review of the literature suggests that females score higher in EI than males [9,10,11]. Women have been found to possess greater emotional knowledge, better emotional awareness, and more interpersonal competencies [12,13]. However, some studies have found no sex differences in EI [14,15,16], or more recent studies have suggested caution regarding sex differences, as they may be mediated by other variables such as age [17]. A third group of studies with adolescents has found that girls show greater Interpersonal EI, but they found no differences in IEI [18]. 

Concerning age, if EI behaves like other intelligences (increasing with age), it should follow a similar course, that is, the higher the cognitive development and social experience, the higher development of EI. Mayer, Salovey, and Caruso [19] claimed that EI appears to increase with age and experience, even in adulthood, finding higher scores in adults than in adolescents.

### 1.3. Emotional Intelligence: Connection with Other Behavioral, Emotional, and Personality Variables

EI has been linked to more: (1) Positive social behaviors (social sensitivity, help-collaboration) [18], cooperation [20], prosocial behaviors [21], behaviors of consideration towards others, self-control, and prosocial leadership [22]; (2) social skills, communication, friends, social and family support [20,23,24,25]; (3) self-control, sociability, self-motivation, and adaptability [26]; (4) empathy [27,28]; (5) positive, constructive conflict resolution [20,27,29,30,31,32,33,34]; (6) anger control in annoying situations [18]; (7) emotional stability [18]; (8) attention to positive emotions versus to stimuli of negative emotions, which provides protection against stress and promotes mental health [35]; and (9) coping with everyday stress [36]. 

In addition, some studies have confirmed that high EI is related to less: (1) Internalizing and externalizing problems [37], internalizing problems (stress, depression, somatic complaints), externalizing problems (aggression, delinquency) [38], and emotional and behavioral problems [39]; (2) hostility and feelings of annoyance, anger [40,41], and anger expression in annoying situations [18]; (3) physical and verbal aggressiveness [27,40,42,43], aggressive behaviors [37], antisocial behavior [44], disruptive behaviors [45], and aggressor behaviors such as self-reported bullying [46]. Moreover, an inverse correlation was found between comprehension and emotion regulation and cybervictimization [47]. People with low EI have shown more behaviors of withdrawal and social anxiety [22]. 

In an adult study [48] comparing a group of patients with various diagnoses (anxiety disorder, mood disorder, substance abuse disorder, psychotic disorder, or borderline personality disorder) with a nonclinical group, the patients were found to have higher levels of attention to feelings, but lower scores on skills to effectively manage their negative emotional states compared to the nonclinical control group. The study provided evidence that EI deficits are related to the presence and severity of clinical symptoms in patients with different mental disorders. In addition, the study of Díaz-Castel et al. [49] showed that adolescents with social anxiety had emotion-regulation difficulties. In this direction, the systematic review of Domínguez-García and Fernández-Berrocal [50] found inverse correlations between comprehension and emotion regulation (TMMS-24) and suicidal behavior. In a similar direction, high scores in comprehension and emotion regulation were inversely associated with depressive symptoms [51]. Additionally, EI scores were negatively associated with problematic Internet and smartphone use and suicidal ideation [52]. Concerning personality traits, several studies have found positive EI relationships with extroversion, openness, agreeableness, and conscientiousness, and negative relationships with neuroticism or emotional instability [53,54,55,56]. Regarding the relationships between EI and self-concept, the study of Guerrero-Barona et al. [15] also confirmed that adolescents with low IEI had a lower physical and social self-concept. In addition, the study of Extremera, Salguero, and Fernández-Berrocal [57] confirmed EI’s relationships with well-being and satisfaction with life; and the study of Reina and Oliva [58] showed the relationship between emotional competencies and two important indicators of adolescent psychological adjustment, self-esteem and life satisfaction. 

Therefore, the results suggest that emotionally intelligent students show better personal and social adaptation, which will help them to better cope with life’s problems. 

### 1.4. Emotional Intelligence: Predictor Variables

Although there is not much research on EI predictors, it has been shown that some personality traits and sociodemographic characteristics can predict it. Among the sociodemographic characteristics, it was found that being female explains EI [59]. Kumar and Bhushan [60] showed that personality (adaptability, sociability, openness, agreeableness, and conscientiousness) predicted self-awareness and self-control within EI. In the same vein, the regression analysis performed by Schulte, Ree, and Carretta [61] showed that personality traits (neuroticism, extroversion, openness, agreeableness, and conscientiousness) predicted EI.

Regarding the connections between EI and family, the results of Chandran and Nair [62] found that the family climate was a predictor of adolescents’ EI. In fact, according to these authors, the relationship with siblings predicted IEI. However, the family climate did not affect interpersonal EI. Likewise, maternal affection was a predictor of general EI. Another study [63] found significant relationships between the perception of parental EI and the family climate perceived by the children. Regression analyses showed that perceived EI is a good predictor of factors such as expressiveness in the family climate.

### 1.5. Goals and Hypotheses

Within this contextualization, this study, which focuses on IEI during adolescence, establishes three objectives: (1) to analyze possible differences in sex, age, and the request for psychological assistance due to various behavioral and emotional problems; (2) to find evidence of the characteristics of adolescents with low IEI related to personality traits (empathy, self-esteem, neuroticism, extroversion, openness, agreeableness, responsibility), social behaviors (antisocial, conflict resolution, bullying/cyberbullying), and parental socialization styles (acceptance-involvement-affection and coercion-discipline-punishment); and (3) to identify the variables that predict high IEI. Based on these objectives, five hypotheses are proposed:

**Hypothesis** **1 (H1).** *During adolescence, no sex differences will be found in IEI (attention, comprehension-clarity, emotion regulation-repair).*

**Hypothesis** **2 (H2).** *IEI (attention, comprehension-clarity, emotion regulation-repair) will increase with age.*

**Hypothesis** **3 (H3).** *Adolescents with a low level of comprehension-clarity and emotion regulation-repair will have consulted a psychologist more frequently during the last year for various behavioral and emotional problems (anxiety, depression, eating problems, school performance, family problems, violence, addictions…).*

**Hypothesis** **4 (H4).** *Adolescents with low IEI (attention, comprehension-clarity, emotion regulation-repair) will have low scores on personality traits (empathy, self-esteem, extroversion, openness, agreeableness, responsibility), will use few cooperative strategies to resolve conflicts, will be more involved in bullying/cyberbullying situations, will present antisocial behaviors, and will have parents with a low level of acceptance-involvement-affection and a high level of coercion-discipline.*

**Hypothesis** **5 (H5).** *The following variables will predict IEI: personality traits such as extroversion, openness, agreeableness, responsibility and neuroticism, cooperative conflict resolution, and parents with positive parental education styles (high acceptance-involvement-affection).*

## 2. Materials and Methods 

### 2.1. Participants

The sample consists of 2283 participants aged 12 to 17 years, from the Basque Country; 1152 (50.5%) males, and 1131 (49.5%) females. Participants were enrolled in Compulsory Secondary Education (CSE), 1180 (51.7%) in the first cycle, and 1103 (48.3%) in the second cycle, and were registered in public (50.2%) and private/concerted (49.8%) schools in the Basque Country. The sample is representative of the CSE students from the Basque Country. According to the population survey submitted by the eustat (eustat.es), the CSE student population was 79,486. With a 99% confidence level, and a sampling error of 0.03 for a population variance of 0.50, the representative sample comprises 2213 subjects. A stratified, proportional, and random sampling technique was used for sample selection, taking into account the proportionality of the schools in each province and the type of school (public/private).

The study sample was collected at 12 public and private schools. Taking as reference the list of schools in the Basque Country, 6 schools of each type were randomly selected. The selection took into account the school population of the 3 provinces of the Basque Country. Samples were collected at each school of all the students in all the classrooms of those age groups, except for 30 students whose parents, or they themselves, declined to participate. The level of rejection of participation was very small.

### 2.2. Procedure

Firstly, a letter was sent to the managers of the selected schools, explaining the research project. With those who agreed to participate, an interview was arranged, and informed consent forms were given to parents/participants. Subsequently, the evaluation tools were administered in two 45-min sessions. The study met the ethical values required in research with humans and received a favorable report from the Commission of Research Ethics of the University of the Basque Country (CEISH/112/212).

### 2.3. Assessment Instruments

To measure the target variables of the study, we used eight assessment instruments with adequate psychometric guarantees of reliability and validity. IEI was measured with the TMMS-24, and 7 complementary instruments were applied to measure other variables (see Table 1).

### 2.4. Data Analysis

Firstly, to explore the existence of differences based on three parameters (sex, age, psychological assistance for different problems in the last year) in Total IEI, as well as in its factors (attention, comprehension, and emotion regulation), descriptive analyses (means and standard deviations) and univariate and multivariate analyses of variance (ANOVA, MANOVA) were performed (after confirming the basic assumptions: Normality, variance homogeneity, etc.), and the effect size was calculated (Cohen’s *d*; partial Eta-squared-$\eta_{p^{2}}$) for the three parameters. Secondly, to analyze the connections between IEI and various personality, behavioral, and family variables, partial correlation coefficients were calculated, taking into account the effect of sex and age. Complementarily, the sample was divided into three groups according to the level of Total IEI (low, medium, and high). Subsequently, descriptive analyses, ANOVA, effect size ($\eta_{p^{2}}$), and group comparisons (Bonferroni) were performed in the three groups and for all the study variables. Effect sizes were interpreted following the standard parameters: Cohen’s *d* (small < 0.50; moderate 0.50–0.79; and large ≥ 0.80) and $\eta_{p^{2}}$ (from 0.01 to 0.04 judged as small, 0.04 to 0.14 moderate, and greater than 0.14 large). Thirdly, to identify variables that predict a high score in Total IEI, step-wise multiple linear regression analysis was performed with the entire sample. In this analysis, the Total IEI (the sum of its three factors) was introduced as a dependent variable, and as predictor variables: personality traits (empathy, self-esteem, neuroticism, extroversion, openness, agreeableness, responsibility), conflict resolution (cooperative, aggressive, passive), bullying (victim, aggressor), cyberbullying (cybervictim, cyberaggressor), antisocial behavior, and family variables (degree of acceptance-involvement-affection and coercion-discipline-punishment of the mother and father). 

## 3. Results

### 3.1. Intrapersonal Emotional Intelligence: Sex Differences 

The results of the MANOVA for all the variables showed differences in Total IEI as a function of sex, Wilks’ Lambda, Λ = 0.939, F(3, 2077) = 44.68, *p* < 0.001, with a small effect size ($\eta_{p^{2}}$ = 0.061, *r* = 0.24). The results of the ANOVA (see Table 2) showed higher scores in IEI in females. However, these differences were mainly due to the differences found in the emotional attention factor, in which significantly higher scores were observed in the females. No sex differences were found either in comprehension (clarity) or emotion regulation (repair), two very relevant factors in IEI. In short, the females presented greater attention capacity or emotion perception, but in comprehension and emotion regulation, both sexes showed similar scores. The effect size was small, both in total IEI and in its factors or dimensions.

### 3.2. Intrapersonal Emotional Intelligence: Age-Related Changes

The results of the MANOVA for all the variables yielded differences depending on the educational cycle (age-related), Wilks’ Lambda, Λ = 0.994, F(3, 2077) = 3.95, *p* < 0.01, although the effect size was very small ($\eta_{p^{2}}$ = 0.006, *r* = 0.07). The results of the ANOVA (see Table 3) showed significant differences between cycles in comprehension (clarity) and emotion regulation (repair), with lower scores in the second cycle, although the effect size was very small. In short, in the first cycle of CSE (12–14 years), the students showed slightly higher scores in comprehension and emotion regulation, compared to the second cycle (15–17 years), which showed a slight decrease in both factors as age increased.

### 3.3. Intrapersonal Emotional Intelligence: Are There Differences between Students Who Requested Psychological Assistance and Students Who Did Not Request It?

The results of the MANOVA for all the variables confirmed differences depending on whether or not the participants had consulted a psychologist for various problems, Wilks’ Lambda, Λ = 0.995, F(3, 2055) = 3.70, *p* <0.01, although the effect size was very small ($\eta_{p^{2}}$ = 0.005, *r* = 0.07). The results of the ANOVAs (see Table 4) yielded no differences in emotional attention and comprehension but differences were found in emotion regulation (repair). In short, students who consulted a psychologist in the last year for various behavioral and emotional problems (anxiety, depression, eating problems, school performance, family problems, problems related to violence, addictions, etc.) obtained significantly lower scores in emotion regulation, although the effect size was very small. 

### 3.4. Intrapersonal Emotional Intelligence: Connection with Personality Traits, Social Behaviors, and Parental Education Styles

The coefficients obtained (see Table 5) showed positive correlations of the three factors of IEI (attention, comprehension, regulation) with: (1) Empathy (Interpersonal Emotional Intelligence); (2) self-esteem (self-assessment); (3) extroversion (sociability, assertiveness, energy and optimism); openness (unconventional, willingness to accept new ideas); agreeableness (altruism, geniality, helping behaviors); responsibility (having the will and ability to achieve objectives); (4) use of cooperative conflict-resolution strategies (based on dialogue and negotiation) and of passive (avoidant) strategies; and (5) a high degree of maternal and paternal affection and acceptance (except for comprehension). Inverse or negative correlations of the three factors were also found with: (1) Neuroticism (emotional instability); (2) antisocial behaviors (except for attention); and (3) aggression and cyberaggression behaviors (except for attention). In addition, negative correlations were found between emotion regulation and the use of aggressive interpersonal conflict-resolution strategies, as well as positive correlations between emotional attention and both parents’ coercion-discipline-punishment.

Therefore, adolescents with high scores in Total IEI and its factors were more likely to have high scores in empathy, self-esteem, extroversion, openness, agreeableness, and responsibility, to use cooperative and passive strategies as an interpersonal conflict-resolution technique, and to have parents with a high level of acceptance-involvement-affection towards their children. In addition, they were more likely to score low in neuroticism, present few antisocial behaviors, and few bullying and cyberbullying behaviors (aggression and cyberaggression).

### 3.5. Personality Traits, Social Behaviors and Family Variables of Adolescents with Low Levels of IEI 

The sample was first divided into three profiles based on the Total IEI level: Profile 1 or low IEI (raw scores 1–63; percentile ≤ 20; *n* = 447; 21.5%); Profile 2 or average IEI (raw scores 64–90; 21–80 percentiles; *n* = 1244; 59.8%); and Profile 3 or high IEI (raw scores 91–120; percentile ≥ 81; *n* = 390; 18.7%). Subsequently, ANOVA and group comparison analyses were performed on all the target variables, the results of which are presented in Table 6. As can be seen, adolescents who had low Total IEI (compared to those with an average or high level) had significantly lower empathy, self-esteem, extroversion, openness, agreeableness, responsibility, use of cooperative and passive interpersonal conflict-resolution strategies, and their parents had a low level of affection, acceptance, and involvement in the lives of their children. These low-IEI adolescents also performed significantly more face-to-face bullying and cyberbullying, and more antisocial behaviors. There were no differences between the three groups in neuroticism, the use of aggressive conflict-resolution strategies, victimization/cybervictimization, or the use of coercion-discipline-punishment by both parents. The effect size was small for many variables (neuroticism, passive/aggresive conflict resolution, bullying/cyberbullying, agreeableness, responsibility, antisocial behavior, mother’s and father’s coercion) and moderate in the rest (empathy, self-esteem, extroversion, openness, cooperative conflict resolution, mother’s affection).

### 3.6. Intrapersonal Emotional Intelligence: Predictor Variables

The results of the Total IEI regression analyses are presented in Table 7. As can be seen, for the total sample, out of the set of Total IEI predictors, six variables were significant: The ability to resolve conflicts cooperatively (β = 0.196), extroversion (β = 225.), responsibility (β = 0.171), openness (β = 0.170), maternal acceptance-affection (β = 0.107), and empathy (β = 0.074). Six variables, which explain 31.5% of the variance, were predictors of IEI: The use of many cooperative conflict-resolution strategies; personality traits such as extroversion, responsibility, openness, and empathy; and a high level of maternal acceptance-affection.

## 4. Discussion

The work studies IEI, with the following objectives: (1) To analyze possible differences due to sex and age, and the request for psychological assistance for behavioral and emotional problems; (2) to seek evidence of personality traits, social behaviors, and parental socialization styles that are characteristic of adolescents with low IEI; and (3) to identify the variables that predict high IEI.

Firstly, the results show that females have greater attention capacity or emotional perception (attention to feelings or ability to feel and identify one’s feelings appropriately) than males, but no sex differences were found in comprehension-clarity (ability to understand one’s own emotional states) or emotion regulation-repair (repairing moods or the ability to adequately regulate moods). These results practically confirm Hypothesis 1, which proposed no sex differences in IEI, although higher scores were found in females’ emotional attention. 

The results point in the same direction as the study with adolescents aged 13–16 years [18] that found no sex differences in IEI, and only higher Interpersonal EI in girls; they also ratify other studies that have found no sex differences [14,15]. However, they contradict other works that have found higher EI in females, although the differences can be explained because, in some of these studies, IEI and interpersonal EI were measured together. Moreover, as other authors have pointed out [17], age may also influence sex differences in EI. The EI variables measured in each study, the instruments used, and the ages of the samples could explain these discrepancies. Several reviewed studies note that sex differences in EI are decreasing in the new generations, and that this change may be due to cultural and educational influences. In another explanatory direction, Fernández-Berrocal, Cabello, Castillo, and Extremera [17] found that the sex differences observed in EI are mediated by age in the facilitation and comprehension branches, in the total score, and, partially, in emotion regulation. These data suggest that it is necessary to be cautious when concluding that sex is decisive in people’s EI, without having examined the possible interactions that other variables may present with sex in the prediction of EI.

Secondly, the results confirm a slight decrease in comprehension-clarity and emotion regulation-repair with age, so Hypothesis 2, which considered an increase in IEI with age, is refuted. These results were unexpected and contradict the findings of other studies finding an increase in EI with age, for example, that of Mayer, Salovey, and Caruso [19]. Differences in the ages of the study samples can partly explain these discrepancies. However, these data emphasize the need to promote the implementation of programs that enhance IEI and Interpersonal EI during childhood and adolescence (see [71,72,73,74,75,76,77,78]).

Thirdly, the work confirms that adolescents who consulted a psychologist in the last year for various problems (anxiety, depression, eating problems, school performance, family problems, problems related to violence, addictions, etc.) had a significantly lower capacity to regulate emotions. The results partially confirm Hypothesis 3, finding less emotion regulation in students who consulted a psychologist, but there were no differences between these two groups in the emotional comprehension ability. These results, in the same direction as other studies, suggest that adolescents with high emotion regulation, a relevant factor of IEI, present fewer symptoms and emotional and behavioral problems. Several studies have confirmed that individuals who have high EI usually show fewer internalizing (anxiety, depression, etc.) and externalizing problems (such as aggression) [37,38,39,49,51]. More symptoms have been observed in people with low EI, both in adolescent and adult studies [48,50].

Fourthly, the results show that adolescents who had low Total IEI (percentiles ≤ 20) had significantly low empathy (ability to consider emotions and the moods of other human beings), low self-esteem (feelings of self-assessment), low extroversion (sociability, assertiveness, optimism), low openness (open, unconventional, willing to accept new ideas), low agreeableness (altruism, geniality, helping behaviors), low responsibility (the will to achieve goals), little use of cooperative interpersonal (based on dialogue and negotiation) and passive (based on avoidance) conflict-resolution strategies; as well as parents with a low level of acceptance, affection, and involvement in the lives of their children. They also performed significantly more face-to-face bullying and cyberbullying, and more antisocial behaviors. The results confirm Hypothesis 4 almost entirely, as no connections were found between IEI and the parents’ level of coercion-discipline-punishment.

These results confirm those obtained in studies, finding positive relationships between EI and the personality traits of extroversion, openness, agreeableness, and conscientiousness [53,54,55,56]. In addition, the results also confirm studies finding that high EI is positively related to empathy [18,27,28], and constructive conflict resolution [20,23,29,30,31,32,33,34], and negatively related to hostility and feelings of anger [40,41], expressions of anger in annoying situations [18], physical and verbal aggression [27,40,42,43], aggressive and antisocial behaviors [18,37,44], and self-reported bullying behaviors [46]. The results also confirm studies finding positive relationships between self-concept, self-esteem, and IEI [15,57,58]. However, no relationships with cybervictimization were found.

The overall effect sizes were small in all variables with regard to sex, age, and psychological assistance. The comparison of profiles with different IEI levels (low, medium, high) also revealed small effect sizes in many variables, and only a moderate effect in a few variables (empathy, self-esteem, extroversion, openness, cooperative conflict resolution, mother’s affection).

Finally, the results identified six predictors of Total IEI: The use of many cooperative conflict-resolution strategies; personality traits such as extroversion, responsibility, openness, and empathy; and a high level of maternal affection-acceptance-involvement. The results largely confirm Hypothesis 5 because low agreeableness and neuroticism were not found among the hypothesized predictors. In this direction, the study of Kumar and Bhushan [60] showed that personality (sociability, openness) predicted self-awareness and self-control within EI; and also the analysis of Schulte et al. [61], which reported that personality (extroversion, openness) predicted EI. In addition, the findings of this study confirm those of Chandran and Nair [62], in which maternal affection was a predictor of general EI. 

Given the importance of EI in human life, it is necessary to identify the factors that help to predict it, because the determination of explanatory variables will be useful in designing programs to develop EI. The work carried out allows us to identify the variables that are relevant to the design of intervention programs aimed at developing EI or variables associated with EI (cooperative conflict resolution, self-esteem, empathy, prosocial behavior, sociability, agreeableness, communication, etc,). Moreover, the study emphasizes the importance of both parents’ affection, involvement, and acceptance, suggesting the importance of training the family in educational guidelines that stimulate children’s EI, which will undoubtedly help them in their personal and social adaptation. Another contribution of the work is to have demonstrated IEI’s connection with bullying/cyberbullying and antisocial behavior. Among the most relevant original contributions of the study are: (1) Having demonstrated the connection between EI and new phenomena of digital violence among adolescents, such as cyberbullying, and in particular, cyberaggression, which emphasizes the relevance of promoting EI as a means of preventing cyber violence among peers; (2) having shown that adolescents with low IEI request psychological help due to the presence of a variety of psychopathological symptoms, which highlights the importance of enhancing EI as a means of promoting mental health; and (3) having identified the relevance of parental affection-involvement-acceptance in IEI, which had not been found in previous studies; in addition to having demonstrated the low weight of both parents’ discipline, punishment, and coercion in their children’s IEI.

Some limitations of the study are the use of self-reports, because of the bias of social desirability they entail, and the cross-sectional nature of the research, which precludes the conclusion of causal connections between IEI and other variables. The results obtained open up future lines of research, including the design, implementation, and evaluation of psychoeducational intervention programs to promote EI, including activities that stimulate the development of the variables identified in this study (self-esteem, sociability, positive conflict-resolution, prosociality, empathy, etc). The assessment of the effects of these programs will provide evidence of the causality of the connections between these variables and EI. Another line of future research could assess the effects of EI-promoting programs on behavioral, emotional, and mental health issues.

The results have practical implications and suggest the importance of promoting EI and socio-emotional development during childhood and adolescence. In general, children attend school from Early Childhood Education, spending a significant portion of their life in the classroom. This period is transcendental due to the physical, intellectual, social, and emotional growth that takes place from these early ages and throughout childhood and adolescence. School is a space where children and adolescents are in contact with their peer group and with situations that generate a diversity of emotions. For this reason, it is considered of great importance to address EI as of early education.

What programs are there to promote EI and socio-emotional development? In order to identify programs published in the last 20 years to be implemented during childhood and adolescence, a review was carried out [79] that identified a large number of programs, describing their objectives, content, techniques and implementation procedures. To mention a few by way of example: JUEGO [GAME] Programs (4–6, 6–8, 8–10, 10–12 years) [80,81,82,83,84], the psychological intervention program with adolescents [85]; the PAHS Program of Assertiveness and Social Skills [86]; the Emotion Education Program [87]; the INTEMO Program, emotional skills for 12- to 18-year-olds [88]; the FORTIUS program, psychological strength and prevention of emotional difficulties [89]; the DULCINEA program for Emotional Education in Secondary Education [90,91]; the PREDEMA emotional education program for adolescents [92]; or the Hero program to stimulate socioemotional development (prosociality, empathy, positive emotions, etc.) [93]. For more details about programs, readers can consult the recent review of Garaigordobil [79].

## 5. Conclusions

The study on IEI in adolescence suggests that: (1) there are small sex differences, as only in emotional attention did females show significantly higher scores; there were no differences in emotional comprehension (clarity) or emotion regulation (repair); (2) between 12 and 17 years, a significant decrease in comprehension (clarity) and emotion regulation (repair) was observed; (3) those adolescents who consulted a psychologist in the last year for various behavioral and emotional problems (anxiety, depression, eating problems, school performance, family problems, violence, addictions, etc.) had significantly less capacity for emotion regulation (repair); (4) adolescents with low IEI (compared to those with an average or high level) showed significantly lower empathy, self-esteem, extroversion, openness, agreeableness, responsibility, use of cooperative and passive conflict-resolution strategies; as well as having parents with a low level of acceptance, affection, and involvement in the lives of their children. Moreover, they performed significantly more bullying, cyberbullying, and antisocial behaviors. (5) the use of cooperative conflict-resolution strategies; personality traits such as extroversion, responsibility, openness, and empathy; and high maternal acceptance-affection, were predictors of high IEI. The work provides information on some relevant variables to take into account for the design of intervention strategies (e.g., empathy, self-esteem, sociability, cooperation, etc.) and makes it possible to conclude that there is a need to promote IEI and interpersonal EI as a factor of development and for the prevention of bullying/cyberbullying.

## Figures and Tables

**Table 1:** Evaluation tools administered.

Instrument	Variables Evaluated	Description of the Test	Psychometric Data
TMMS 24. Trait Meta-Mood Scale [7,14]	Perceived (Intrapersonal) Emotional Intelligence (IEI); Emotional attention (Attention); Emotional comprehension (Clarity); Emotion regulation (Repair)	Self-report comprised of 24 items (8 for each factor), and participants rate their agreement on a 5-point Likert-type scale, ranging from 1 (*strongly disagree)* to 5 (*strongly agree*)	Reliability: Attention (α = 0.90); Comprehension (α = 0.90); Regulation (α = 0.86). Validity: Positive correlations have been shown with life satisfaction, and negative correlations with depression and ruminative responses
QMEE. Questionnaire Measure Emotional Empathy [64]	Empathy; Ability to understand other people’s emotions	Self-report of 22 items: participants indicate whether they do, think or feel what is expressed in the phrase	Reliability: α = 0.74. Validity: High correlations with empathy
RSE. Rosenberg Self-Esteem Scale [65]	Self-esteem; Global feelings of self-assessment	Self-report with 10 statements: Participants rate their agreement with the statements on a 3-point Likert scale ranging from 1 (*strongly agree*) to 3 (*strongly disagree*)	Reliability: α = 0.77; Test-retest reliability: *r* = 0.85. Validity: Positive correlations with self-esteem
NEO-FFI. Five-Factor Reduced NEO Personality Inventory [66]	Big-5 personality dimensions: Neuroticism, Extroversion, Openness, Agreeableness, Responsibility	Self-report with 60 statements: participants rate their agreement with the content of the sentence, ranging from 0 (*Completely disagree*) to 4 (*Completely agree*)	Reliability: Neuroticism (α = 0.90); Extroversion (α = 0.84); Openness (α = 0.82); Agreeableness (α = 0.83); and Responsibility (α = 0.88). Validity: Factorial rotation to maximize the convergent and discriminant validity of validemex factors of the NEO-PI
CONFLICTALK. Questionnaire to measure the message styles in conflict management [67]	Conflict-Resolution Style: Cooperative, passive-avoidant, aggressive	Participants rate the extent to which they perform or say the expressions of 18 phrases in a conflict situation, on a scale ranging from 1 (*never*) to 5 (*usually*).	Reliability: Cooperative Resolution (α = 0.87), Aggressive Resolution (α = 0.81), Avoidant Resolution (α = 0.63) .Validity: Positive correlations between communication skills and cooperative resolution, and negative ones with aggressive and avoidant resolution
CYBERBULLYING. Peer bullying screening. Screening of face-to-face school bullying and technological bullying (cyberbullying) [68]	Bullying: Victimization, Aggression, Observation, Cyberbullying, Cybervictimization, Cyberaggression, Cyberobservation	Self-reporting on 4 face-to-face bullying behaviors and 15 cyberbullying behaviors: Participants report how often they have suffered, performed or observed them in the last year	Bullying: Total reliability (α = 0.81), Victimization (α = 0.70), Aggression (α = 0.71), Observation (α = 0.80). Cyberbullying: Total reliability (α = 0.91), Cybervictimization (α = 0.82), Cyberperpetration (α = 0.91), and Cyberobservation (α = 0.87). Validity: Negative correlations with empathy, social adaptation, and positive ones with antisocial behavior
AD. Antisocial-Delinquent Behavior Questionnaire [69]	Antisocial Behavior; Transgression of social norms	Self-report 20 items: Participants report whether the behavior was performed in the last year	Reliability: α = 0.86; Criterial validity: adolescents with behavioral problems scored higher than control adolescents
ESPA29. Scale of Parental Socialization in Adolescence [70]	Parental socialization style of father and mother: Degree of acceptance-involvement-affection; Degree of coercion-discipline-punishment.	Participants assess their father’s and mother’s performance independently in 29 relevant situations	Reliability: α ≥ 0.70; Validity: Factorial analysis confirmed the theoretical structure of the two-dimensional model.

**Table 2:** Means, standard deviations, results of the ANOVA of IEI as a function of sex, and effect size (Cohen’s *d*).

	Male	Female	F(1, 2281)	*p*	*d*
	M (SD)	M (SD)			
Attention	23.32 (7.12)	26.34 (7.15)	93.34	≤0.001	−0.42
Comprehension	25.18 (6.65)	25.01 (6.99)	0.32	0.567	−0.02
Regulation	26.26 (6.64)	26.30 (6.82)	0.01	0.909	−0.00
Total IEI	74.76 (16.67)	77.65 (17.01)	15.25	≤0.001	−0.17

**Table 3:** Means, standard deviations, results of the ANOVA in IEI depending on the educational cycle, and effect size (Cohen’s *d*).

	CSE 1	CSE 2	F(1, 2281)	*p*	*d*
	M (SD)	M (SD)			
Attention	24.78 (7.42)	24.92 (7.15)	0.18	0.666	0.00
Comprehension	25.44 (7.24)	24.75 (6.37)	5.35	0.021	0.10
Regulation	26.64 (7.03)	25.92 (6.40)	6.06	0.014	0.10
Total IEI	76.86 (18.20)	75.58 (15.48)	2.98	0.084	0.07

Note: CSE = Compulsory Secondary Education. CSE 1 = First Cycle; CSE 2 = Second Cycle.

**Table 4:** Means, standard deviations, and results of the ANOVA of IEI, based on psychological assistance, and effect size (Cohen’s *d*).

	Psychological Assistance (*n* = 391)	No Psychological Assistance (*n* = 1668)	F(1, 2281)	*p*	*d*
	M (SD)	M (SD)			
Attention	25.18 (7.95)	24.78 (7.14)	0.93	0.335	0.05
Comprehension	25.24 (7.53)	25.05 (6.64)	0.25	0.616	0.02
Regulation	25.64 (7.33)	26.44 (6.56)	4.54	0.033	0.11
Total IEI	76.05 (18.97)	76.27 (16.37)	0.52	0.819	0.01

**Table 5:** Correlations between IEI and personality traits, social behaviors, and family variables.

	Attention	Comprehension	Regulation	Total IEI
Empathy	0.29 (0.000)	0.16 (0.000)	0.21 (0.000)	0.27 (0.000)
Self-esteem	0.07 (0.001)	0.25 (0.000)	0.33 (0.000)	0.27 (0.000)
Neuroticism	−0.14 (0.000)	−0.14 (0.000)	−0.21 (0.000)	−0.08 (0.000)
Extroversion	0.20 (0.000)	0.25 (0.000)	0.31 (0.000)	0.31 (0.000)
Openness	0.34 (0.000)	0.19 (0.000)	0.22 (0.000)	0.31 (0.000)
Agreeableness	0.17 (0.000)	0.20 (0.000)	0.32 (0.000)	0.28 (0.000)
Responsibility	0.19 (0.000)	0.27 (0.000)	0.35 (0.000)	0.33 (0.000)
Passive Resolution	0.31 (0.000)	0.26 (0.000)	0.27 (0.000)	0.35 (0.000)
Aggressive Resolution	0.02 (0.330)	0.01 (0.710)	−0.05 (0.014)	−0.01 (0.675)
Cooperative Resolution	0.14 (0.000)	0.08 (0.000)	0.06 (0.003)	0.12 (0.000)
Victimization	0.05 (0.022)	−0.01 (0.511)	−0.03 (0.119)	−0.01 (0.932)
Aggression	−0.03 (0.165)	−0.05 (0.010)	−0.10 (0.000)	−0.07 (0.001)
Cybervictimization	0.07 (0.001)	0.01 (0.442)	−0.01 (0.927)	0.03 (0.098)
Cyberaggression	−0.03 (0.101)	−0.06 (0.003)	−0.08 (0.000)	−0.07 (0.001)
Antisocial Behavior	−0.01(0.382)	−0.06 (0.002)	−0.13 (0.000)	−0.09 (0.000)
Mother’s Affection	0.16 (0.000)	0.22 (0.000)	0.22 (0.000)	0.25 (0.000)
Father’s Affection	0.12 (0.000)	−0.01 (0.704)	0.19 (0.000)	0.20 (0.000)
Mother’s Coercion	0.07 (0.010)	0.16 (0.000)	0.02 (0.420)	0.03 (0.206)
Father’s Coercion	0.06 (0.028)	−0.01 (0.700)	0.03 (0.202)	0.03 (0.194)

**Table 6:** Means, standard deviations of all the variables in the three profiles of IEI, ANOVA according to the profile, effect size, and comparison of groups (Bonferroni).

	Profile 1 (*n* = 447)	Profile 2 (*n* = 1244)	Profile 3 (*n* = 390)	F (2, 2210) Profile	$\eta_{p^{2}}$	Post-Hoc
	M (SD)	M (SD)	M (SD)			
Empathy	14.44 (4.28)	16.22 (3.49)	17.40 (3.28)	71.42 ***	0.065	1 < 2 < 3
Self-esteem	27.77 (4.48)	29.69 (5.00)	31.48 (6.00)	54.86 ***	0.051	1 < 2 < 3
Neuroticism	20.94 (6.81)	20.78 (7.21)	19.87 (8.88)	2.44 ns	0.003	---
Extroversion	30.26 (6.70)	33.40 (6.43)	36.71 (6.79)	93.41 ***	0.088	1 < 2 < 3
Openness	22.40 (6.20)	24.86 (6.31)	28.81 (7.44)	95.01 ***	0.089	1 < 2 < 3
Agreeableness	26.63 (5.22)	28.81 (5.62)	31.77 (6.54)	78.22 ***	0.074	1 < 2 < 3
Responsibility	24.66 (6.46)	28.03 (6.35)	31.46 (7.78)	100.76 ***	0.094	1 < 2 < 3
Passive Resolution	10.97 (3.51)	11.74 (3.58)	12.29 (4.43)	13.09 ***	0.013	1 < 2 < 3
Aggressive Resolution	10.00 (3.50)	9.99 (3.69)	9.75 (3.92)	0.66 ns	0.001	---
Cooperative Resolution	13.07 (5.22)	15.75 (5.58)	19.14 (6.54)	115.69 ***	0.102	1 < 2 < 3
Victimization	0.79 (1.36)	0.79 (1.37)	0.81 (1.46)	0.04 ns	0.000	---
Aggression	0.91 (1.55)	0.73 (1.25)	0.62 (1.13)	5.25 **	0.005	1 > 2.3
Cybervictimization	0.83 (2.29)	0.80 (2.12)	0.98 (2.11)	0.97 ns	0.001	---
Cyberaggression	0.83 (3.66)	0.41 (1.70)	0.36 (1.20)	6.64 ***	0.006	1 > 2.3
Antisocial Behavior	7.76 (5.41)	7.33 (5.26)	6.18 (5.46)	9.90 ***	0.009	1.2 > 3
Mother’s Affection	3.12 (0.40)	3.28 (0.38)	3.44 (0.41)	34.59 ***	0.058	1 < 2 < 3
Father’s Affection	3.06 (0.47)	3.21 (0.45)	3.34 (0.52)	20.00 ***	0.034	1 < 2 < 3
Mother’s Coercion	1.78 (0.39)	1.78 (0.38)	1.83 (0.43)	1.26 ns	0.002	---
Father’s Coercion	1.78 (0.45)	1.78 (0.44)	1.82 (0.47)	0.53 ns	0.001	---

Notes: Profile 1 or low IEI (percentile ≤ 20), Profile 2 or average IEI (percentile 21–80), Profile 3 or high IEI (percentile ≥ 81); $\eta_{p^{2}}$ = partial Eta-squared (effect size); Post-Hoc = Bonferroni. * *p* < 0.05. ** *p* < 0.01. *** *p* < 0.00. ns = nonsignificant.

**Table 7:** Predictor variables of Total IEI.

Variables	R	R^2^	ΔR^2^	B	Error	Constant	β	t
Cooperative Resolution	0.369	0.136	0.135	0.539	0.07	59.87	0.196	6.92 ***
Extroversion	0.468	0.219	0.217	0.528	0.06	39.36	0.225	8.29 ***
Responsibility	0.516	0.266	0.264	0.407	0.06	28.38	0.171	6.26 ***
Openness	0.545	0.297	0.295	0.402	0.06	22.29	0.170	6.02 ***
Mother’s Acceptance-Affection	0.557	0.310	0.307	4.225	1.10	10.02	0.107	3.82 ***
Empathy	0.561	0.315	0.311	0.323	0.12	9.11	0.074	2.53 **

Note: ** *p* < 0.01. *** *p* < 0.001.
